# Tumor-Targeted Fluorescent Proteinoid Nanocapsules Encapsulating Synergistic Drugs for Personalized Cancer Therapy

**DOI:** 10.3390/ph14070648

**Published:** 2021-07-06

**Authors:** Ella Itzhaki, Elad Hadad, Neta Moskovits, Salomon M. Stemmer, Shlomo Margel

**Affiliations:** 1 Department of Chemistry, Institute of Nanotechnology and Advanced Materials (BINA), Bar-Ilan University, Ramat-Gan 5290002, Israel; elaeli3543@gmail.com (E.I.); eladha300@gmail.com (E.H.); 2 Davidoff Center, Rabin and Felsenstein Medical Center, Beilinson Campus, Petach Tikva 49100, Israel; neta.moskovits@gmail.com (N.M.); stemmer@post.tau.ac.il (S.M.S.); 3 Sackler Faculty of Medicine, Tel Aviv University, Tel Aviv 6997801, Israel

**Keywords:** personalized cancer therapy, proteinoids, RGD polymers, synergistic drugs, nanocapsules, targeted delivery

## Abstract

Personalized cancer treatment based on specific mutations offers targeted therapy and is preferred over “standard” chemotherapy. Proteinoid polymers produced by thermal step-growth polymerization of amino acids may form nanocapsules (NCs) that encapsulate drugs overcoming miscibility problems and allowing passive targeted delivery with reduced side effects. The arginine-glycine-glutamic acid (RGD) sequence is known for its preferential attraction to αvβ3 integrin, which is highly expressed on neovascular endothelial cells that support tumor growth. Here, tumor-targeted RGD-based proteinoid NCs entrapping a synergistic combination of Palbociclib (Pal) and Alpelisib (Alp) were synthesized by self-assembly to induce the reduction of tumor cell growth in different types of cancers. The diameters of the hollow and drug encapsulating poly(RGD) NCs were 34 ± 5 and 22 ± 3 nm, respectively; thereby, their drug targeted efficiency is due to both passive and active targeting. The encapsulation yield of Pal and Alp was 70 and 90%, respectively. In vitro experiments with A549, MCF7 and HCT116 human cancer cells demonstrate a synergistic effect of Pal and Alp, controlled release and dose dependence. Preliminary results in a 3D tumor spheroid model with cells derived from patient-derived xenografts of colon cancer illustrate disassembly of spheroids, indicating that the NCs have therapeutic potential.

## 1. Introduction

For decades, cancer has been a leading cause of death and has had a major impact on public health and the economy [1,2]. Development of cancer requires multiple changes that drive progressive and metastatic transformation of cells [3]. Many cancers may be treated, and the survival rate has been continually rising owing to early detection and effective therapeutic approaches [4] especially in high income countries [1,2].

Personalized medicine is an approach to patient care based on specific mutations, which has an advantage over “standard treatment” (e.g., chemotherapy usually given for the indication, stage, etc.) as it enables us to maximize efficacy by targeting the right drug or drug combination to the right patient at the right time. In the metastatic stage of solid tumors, development of drug resistance poses a major challenge, which may be overcome by combination therapy [5]. The main drawback of combination therapy is the potential of increased side effects [6]; a conventional application of combined drugs often suffers from limited effectiveness, poor biodistribution, lack of selectivity and toxicity [7,8,9,10]. These drawbacks of free drugs can be overcome by entrapping the drugs within various biocompatible carriers such as nanoparticles, nanocapsules, liposomes, niosomes or polymersomes.

Proteinoids are polymers prepared by thermal step-growth polymerization of amino acids. According to the procedure developed by Fox and Harada [11,12,13], preparation of proteinoids begins by heating specific amino acids (at ~180 °C) in an inert atmosphere. Aspartic acid serves as a solvent for the other amino acid monomers; under heating it condenses to cyclic 2,2′-(3,6-dioxopiperazine-2,5-diyl) diacetic acid, which initiates polymerization with the other amino acids, forming an acidic proteinoid polymer [14,15,16,17]. Proteinoids are made of natural building blocks and, therefore, resemble proteins and are biocompatible, non-toxic and non-immunogenic [18,19]. They can fold in aqueous solution via a self-assembly process that involves dissolution by heating and slow cooling to form hollow nanocapsules (NCs). Suitable molecules can be encapsulated within the internal core of the formed particle owing to hydrophobic residues; a marker (e.g., a fluorescent label) can be conjugated to the surface using hydrophilic residues such as amines [20]. Previous studies presented the encapsulation of various molecules for cosmetics (retinoic acid [21]), chemotherapeutics (doxorubicin and Paclitaxel [18,22,23], cannabidiol [24]), antipsychotics (Risperidone [25]) and dyes for diagnostic imaging (ICG [26,27]).

D-arginine (R), glycine (G) and L-aspartic acid (D) were used for randomly achieving the RGD sequence within the proteinoid backbone; previous studies showed that 13% of the established proteinoids contain the RGD sequence [26,28]. The R^D^GD polymer configuration denoted here as P(RGD) is the polymer of choice for targeted drug delivery to areas of angiogenesis such as a tumor microenvironment [28]. The RGD sequence is known for its specific attraction to αvβ3 integrin, an endothelial cell receptor for the extracellular matrix, which is highly expressed on neovascular endothelial cells that support tumor growth [29,30]. Solid tumors depend on the ability to generate new blood vessels (angiogenesis) for adequate supply of blood to the growing tumor, therefore RGD-based proteinoids are expected to selectively target the tumor microenvironment.

The use of RGD NCs confers several advantages: (i) the small size (20–150 nm) leads to “passive targeting” of tumors via the so-called enhanced permeability and retention (EPR) effect [31]; (ii) due to the relatively small diameter, renal filtration is avoided, leading to prolonged blood circulation and longer accessibility of the ligand to target receptors within the tissue [32]; (iii) RGD targeted nanocarriers may specifically address drugs to angiogenic endothelial cells and/or cancer cells by binding the RGD peptide to αvβ3 overexpressed by these cells, allowing “active targeting” of the tumors [33]; and (iv) RGD targeted nanocarriers can be internalized via receptor-mediated endocytosis, which is not possible with single peptide constructs or non-targeted nanocarriers; this is particularly interesting for intracellular delivery of drugs to cancer cells [34].

**Figure 1:**
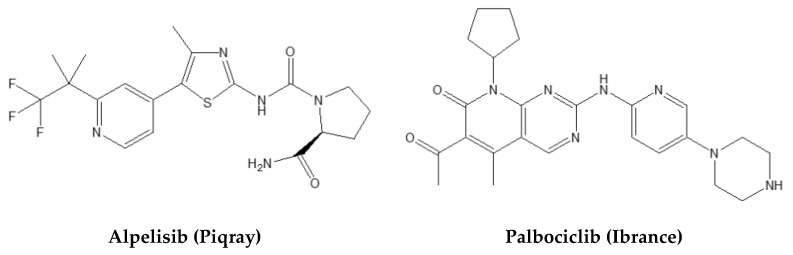
Molecular structures of Alpelisib (Alp) and Palbociclib (Pal).

Here, P(RGD) NCs were used to prove the concept of nanomedicine-mediated multimodal treatment by co-encapsulation of Palbociclib (Pal) and Alpelisib (Alp), commercially known by the brand names Piqray and Ibrance, respectively (Figure 1). Pal is a small molecule that targets and potently inhibits CDK4/6, which plays a vital role in proliferation of mammalian cells [35], as well as cyclin-D1 activities [36]. Its most common side effects are diarrhea, febrile neutropenia, anemia, leukopenia, fatigue, and nausea [37,38,39,40]. Alp is a small orally bioavailable selective inhibitor of P13K signaling that specifically targets PI3Kα associated with cell growth, proliferation, and motility of tumor cells [41]. The most common side effects of Alp are gastrointestinal disorders, hyperglycemia, fatigue, and rash [42]. Their combination thus has a good rationale and holds promise for improving the long-term prognosis and increasing the therapeutic index.

We hypothesized that encapsulating the drugs in RGD-based nanocapsules (P(RGD) NCs) would result in selective delivery to the place of action, such that their influence on vital tissues and undesirable side effects would be minimized. The delivery system also protects the drugs from rapid degradation or clearance and thus enhances their concentration in target tissues, thereby potentially allowing lower doses.

P(RGD) proteinoids were synthesized, characterized in terms of molecular weight and polydispersity, and self-assembled to obtain hollow and drug-encapsulating NCs. An in vitro study was then performed using colon cancer cells derived from patient-derived xenografts (PDX), a model in which a portion of (preferably) fresh tumor is implanted directly in immunocompromised mice [43]. The combination of Pal and Alp was found to have a significant synergistic effect that induces reduction of tumor growth [44]. 

Three-dimensional (3D) cultures using patient tumor-derived cells were chosen as an alternative in vitro model, as in this model the context and conditions closely resemble in vivo conditions, including a network of cell-to-cell and cell to extracellular matrix (ECM) interactions, and better mimic the native tumor microenvironment. Development of a 3D culture offers a bridge between a traditional 2D in vitro culture comprising a monolayer of cells expanded on a flat surface and in vivo experiments [45,46]. Eventually, the spheroids assume a structure that mimics the solid tumor with an outside layer of proliferating cells (high oxygen and nutrient exposure) and an inner layer with necrotic core cells (less oxygen and nutrients) [47].

The present article describes the synthesis and characterization of hollow and drug loaded poly(RGD) NCs entrapping a synergistic combination of the cancer drugs Pal and Alp in order to induce reduction of tumor cell growth in different types of cancers. 

## 2. Results and Discussion

### 2.1. Synergistic Effect of Free Pal and Alp In Vitro

**Figure 2:**
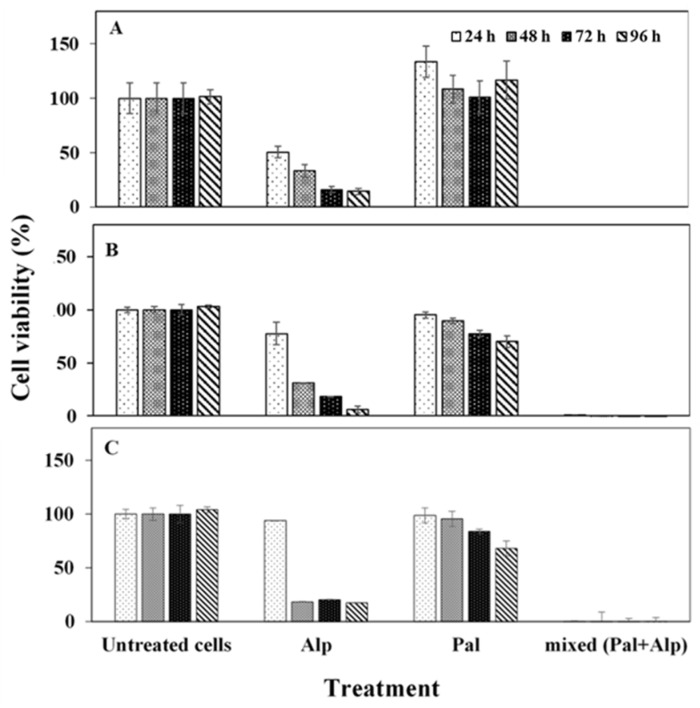
Viability of MCF-7 (**A**), HCT116 (**B**) and A549 (**C**) cells after exposure to Pal/Alp or Pal+Alp (2.8 µM of each drug) measured by XTT assay. Untreated positive control containing only medium and treated cells were incubated for 24, 48, 72 or 96 h. Each bar represents mean ± standard deviation of six separate samples.

To evaluate possible synergy of Pal and Alp, in vitro cytotoxicity was assessed by the XTT assay using MCF-7 (breast adenocarcinoma), HCT116 (colon carcinoma) and A540 (lung carcinoma) human cancer cells. These cell lines have mutations in CDKN2A and PIK3CA [48], which are targeted by Pal and Alp, respectively [49]. The XTT colorimetric assay quantifies the conversion of tetrazolium salts into formazan dye (photometrically detectable), which is reduced by the metabolic ability of active cells. Figure 2 exhibits the cell viability levels post treatment. Pal had no significant effect on cell viability, while Alp demonstrated reduced viability after 48–96 h. Importantly, the combination of Pal and Alp had a significant effect, resulting in a complete eradication of the tumor cells already after 24 h. These findings indicate the significant synergistic effect of combined drugs compared to mono treatment, encouraging development of an effective procedure for synthesis of drug loaded NCs.

### 2.2. Synthesis and Characterization of P(RGD)/Pal,Alp NCs

Proteinoid NCs containing both drugs were prepared similar to the hollow NCs, as described in the experimental section. In order to obtain maximal encapsulation for therapeutic anticancer activity, various drugs/protenoid weight ratios (1, 5, 10, 25 and 50) were used; in most cases, Tween 80 surfactant was added to obtain a clear solution (see Table 1).

**Figure 3:**
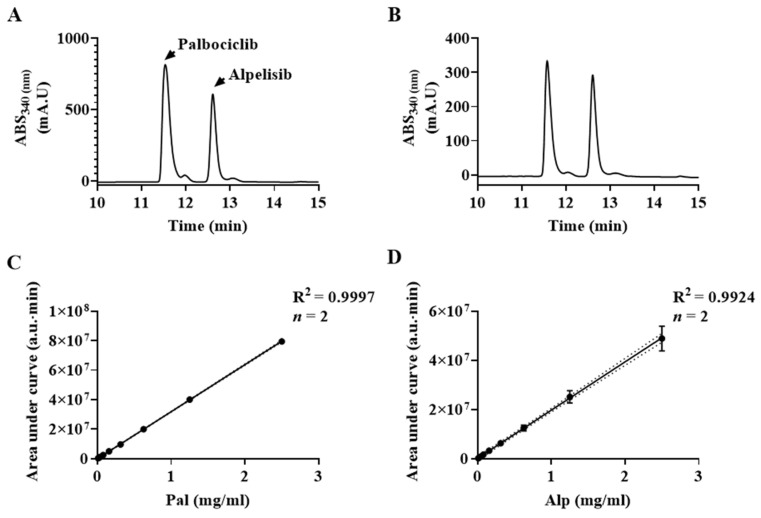
Determination of encapsulated drug concentrations in P(RGD)/Pal,Alp NCs. (**A**) Chromatogram of 2.5 mg/mL standard solution (1.25 mg/mL of each drug); drugs were mixed together to resemble the target sample matrix. The retention time (RT) was 11.5 and 12.6 min for Pal and Alp, respectively. (**B**) Chromatogram of a representative sample of encapsulated drugs analyzed after sonication. RTs were 11.6 and 12.6 min for Pal and Alp, respectively. (**C**,**D**) Standard calibration curves of Pal and Alp (*n* = 3).

The drugs/NCs mixtures were analyzed by HPLC for their drug loading (DL) capacity using calibration standard solutions of Pal and Alp (Figure 3) [50]. The mixtures were also characterized by HR-SEM for their dry diameter (see Figure S1B in the Supporting Information) and by cryo-TEM for their hydrodynamic diameter (see Table 2 and Figure S1C,D). Overall, the dry diameter increased with the total encapsulated drug concentration (Figure S1A), while the wet diameter decreased (explained below in the cryo-TEM results section). The loaded NC wet diameters ranged from 22 ± 3 to 40 ± 8 nm.

In conclusion, we decided to continue with 25% mixed drugs/NCs (50% yielded only a slightly higher DL, see Table 2). These NCs were characterized further by cryo-TEM for hydrodynamic diameter, FTIR, TGA and ζ-potential measurements.

### 2.3. Hydrodynamic Diameter and Size Distribution of Hollow and Drug Loaded NCs

**Figure 4:**
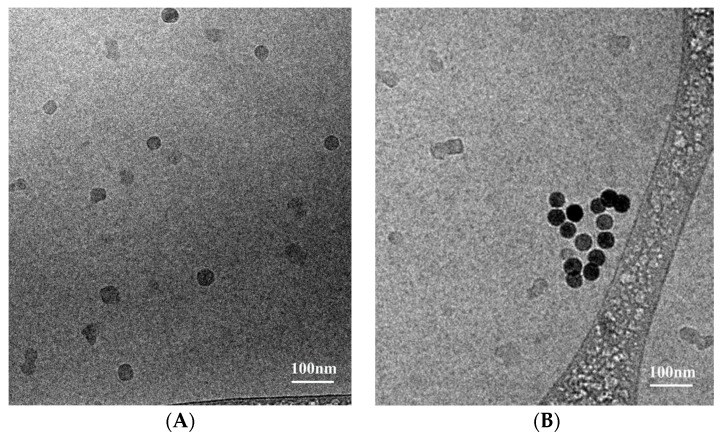
Cryo-TEM images of (**A**) hollow P(RGD) and (**B**) P(RGD)/Pal,Alp NCs.

The hollow and 25% drug-loaded NCs demonstrated a hydrodynamic diameter of 34 ± 5 and 22 ± 3 nm, respectively, as shown in Figure 4 and Table 2. The dry diameter (HR-SEM) is commonly much lower than the hydrodynamic diameter due to hydrated water layers adsorbed onto the particle surface, in addition to water molecules entrapped within the particles [25,28], e.g., iron oxide nanoparticles prepared in our laboratory possessed average dry and hydrodynamic diameters of 18 and 70 nm, respectively [51]. Similarly, bisphosphonate nanoparticles had average dry and hydrodynamic diameters of 43 and 160 nm, respectively [23]. Notably, here the dry diameter of NCs containing 25% mixed drugs/proteinoid (113 ± 31 nm) was also significantly higher than the hydrodynamic diameter (22 ± 3 nm). This may be explained by the presence of part of the drugs on the surface of the NCs, preventing water molecules from being entrapped and adsorbed due to the hydrophobic nature. Further evidence for such drug surface adsorption was provided by the increasing difference in the dry and hydrodynamic diameters with increasing initial weight ratio (drugs)/(proteinoid); for example, in the absence of drugs, both diameters are similar, while at 1, 5 and 50% loading, the difference between the two diameters increased to 29, 55 and 108 nm, respectively, as shown in Table 2. Moreover, we should emphasize that cryo-TEM tomography provides a more reliable measure as it also considers the four dimensionality (4D). Cryo-TEM was also used to quantitate the wet diameter and drug content. Hollow NCs are lightly shaded, while those with drugs are darker, confirming that the active drugs are indeed entrapped [52] (cf. Figure 4A,B).

### 2.4. ζ-Potential Measurements

**Figure 5:**
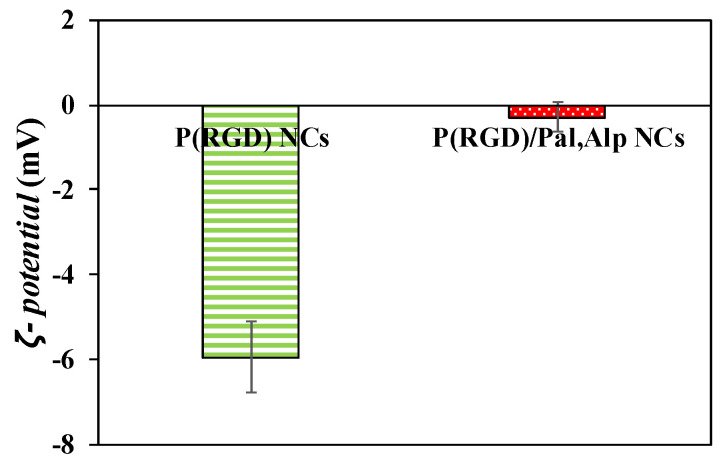
ζ-potentials of P(RGD) and P(RGD)/Pal,Alp NCs.

To examine the hypothesis that drugs reside on the surface, the zeta potentials of the NCs were measured. The ζ-potential of a NC in a dispersed medium is related to the electro-potential in that liquid environment. Potentials were measured at a pH of 7.5 as described in the methods section, yielding −6 ± 2 and −0.3 ± 0.8 mV for hollow and drug loaded NCs, respectively (Figure 5). The negative charge is due to the carboxyl residue of aspartic acid on the NC surface [26]. The increase corresponds well with the DL and the physical interactions of the drugs with the surface, which mask the negative residue.

### 2.5. FTIR Analysis of the Free Drugs and P(RGD) Proteinoid NCs

Pal, Alp and the P(RGD) proteinoid NCs were characterized by FTIR (Figure S2). The spectrum of Alp (Figure S2A) demonstrates typical peaks at 1400–1000 cm^−1^ corresponding to C–F vibration, 1317 cm^−1^ (C–S stretching band [53]), 1640 cm^−1^ (carbonyl stretching band), broad peaks at 2770–2870 cm^−1^ (C-H and CH_3_ groups), and a peak at 3500 cm^−1^ corresponding to N–H stretching band [54]. The spectrum of Pal (Figure S2B) demonstrates typical peaks at 1900–1640 cm^−1^ corresponding to the carbonyl stretching band, 2780–2960 cm^−1^ (C–H and CH_3_ stretching band [55]) and 3500 cm^−1^ (N–H stretching band [54]). The spectrum of P(RGD) (Figure S2C) demonstrates typical peaks of proteinoids [21,50] at 660 and 770 cm^−1^ (amine N-H wagging [56]) belonging to the arginine side chain, which possesses three atoms of nitrogen. The peaks at 1050 and 1200 cm^−1^ correspond to the C-N stretching band, the peak at 1560 cm^−1^ corresponds to amide N-H stretching band, and the broad peak at 2700–3500 cm^−1^ can be attributed to C-H, N-H and O-H stretching bands [32]. The spectrum of P(RGD)/Pal,Alp proteinoid NCs (Figure S2D) shows peaks that can be attributed to both the drugs (Alp and Pal) and the NCs.

### 2.6. Thermogravimetric Analysis (TGA)

The thermal behavior of Pal, Alp, and hollow and drug-loaded proteinoid NCs was investigated by thermogravimetric analysis (TGA, Figure S3). The thermogram of Alp shows two decomposition slopes, first at 200–350 °C with 90% weight loss and a second slope at 400–470 °C, with 10% weight loss of Alp. The thermogram of Pal shows two decomposition slopes: at 275–400 °C with 50% weight loss, followed by a slope at 500–640 °C with 40% weight loss of Pal. The thermogram of the P(RGD) NCs presents three decomposition slopes: at 25–200 °C with 15% weight loss, at 220–550 °C with 55% weight loss, and at 575–600 °C with 30% loss. The P(RGD)/Pal,Alp NCs exhibit five decomposition slopes: at 130–150 °C similar to the first slope of the hollow NCs, at 200–240 °C, a third slope at 260–320 °C, comparable to the first decomposed slope of Alp and Pal; the slope at 350–500 °C and the fifth, more moderate slope at 550–610 °C, are both similar to the slopes of Pal and the hollow NCs. The thermal stability of NCs encapsulating mixed drugs increased at 60 °C compared to the hollow NCs, possibly due to hydrophobic interactions or hydrogen bonds between the drugs and the proteinoid that strengthen the NC. 

### 2.7. Conjugation of Cy7 to NCs and Drugs Loading of Pal and Alp

**Figure 6:**
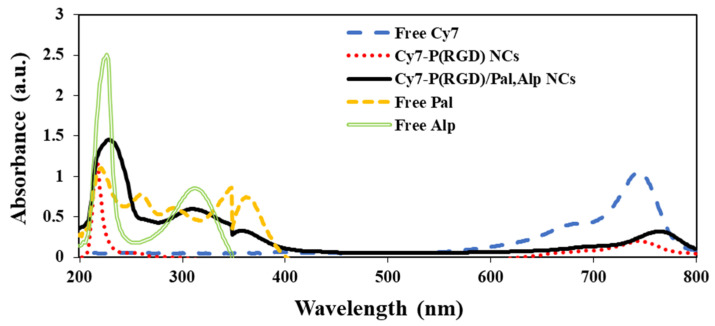
Absorbance spectra of free Cy7 (blue light-dashed line), Cy7-conjugated P(RGD) NCs (red dotted line), Pal (orange dashed line), Alp (green hollow line) and Cy7-conjugated P(RGD)/Pal,Alp NCs (black solid line).

**Figure 7:**
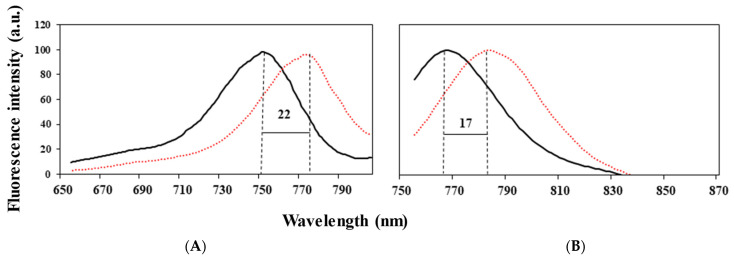
Fluorescence intensity shift of P(RGD)/Pal,Alp NCs. (**A**) Excitation and (**B**) emission peaks for free Cy7 (black line) and Cy7-conjugated NCs (orange dotted line).

Here, Cyanine7 NHS ester (Cy7) near infrared (NIR) dye (600–900 nm) was used to minimize the background problems of high autofluorescence and tissue penetration [57]. The Cy7 conjugation yield in the hollow and drug-encapsulating P(RGD) NCs was determined using a calibration curve of free Cy7 in water as described in the experimental section. The Cy7 conjugation yield was 100%, meaning that the Cy7-conjugated NCs contain 1% of the dye (relative to the NCs). Figure 6 shows the UV-Vis absorption spectra of the Cy7-P(RGD)/Pal,Alp NCs compared to the Cy7-conjugated hollow NCs, free Cy7, and free Pal and Alp. It can be clearly seen that the Cy7 dye and Pal and Alp drugs were successfully conjugated/encapsulated to the NCs, indicated by the absorbance at 600–800, 315 and 340 nm, respectively. The shift in the peak is probably due to the conjugation of Cy7 to the different NCs [58,59]. Hollow and drug loaded NCs both show the characteristic peak of proteinoids at 218 nm. Figure 7B establishes the shift in the emission peak of free Cy7 from 766 to 783 nm for the P(RGD)/Pal,Alp Cy7-conjugated NCs, and the shift in the excitation peak from 750 to 774 nm is shown in Figure 7A. The shift in fluorescence is probably due to encapsulation within the small interior space of the NCs, which results in aggregation of dye molecules [33,34].

### 2.8. Photostability of Encapsulated NCs and Free Pal

**Figure 8:**
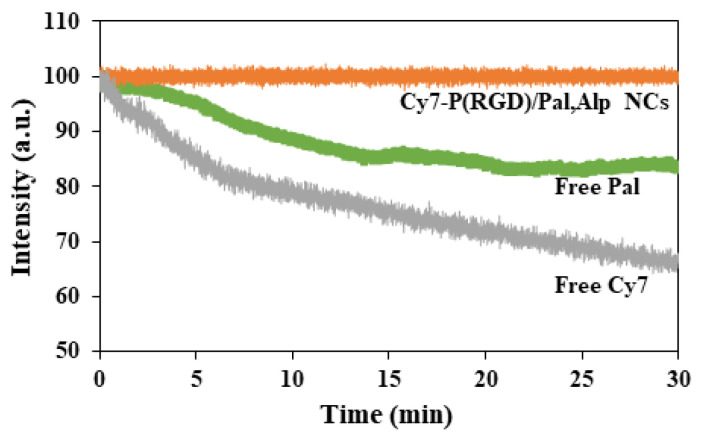
Photostability of Cy7-P(RGD)/Pal,Alp NCs (orange line), free Cy7 (gray line) and free Pal (green line) as a function of time. Fluorescence intensities were recorded after illumination with a xenon flash lamp for 30 min.

Photobleaching is a major limitation of cyanine dyes. Upon illumination of aqueous solutions of Cy7, reactive oxygen species such as peroxide, superoxide and redox active metabolites can react with fluorophores and lead to their degradation [60]. In order to examine the protection of the conjugation of Cy7 to P(RGD)/Pal,Alp NCs, photobleaching experiments were performed for both encapsulated and free dyes. Samples of free and NC-conjugated Cy7 were illuminated over a period of 30 min. During illumination, the fluorescence intensity of the Cy7-P(RGD)/Pal,Alp NCs was maintained, while the intensity of Cy7 decreased, as shown in Figure 8. Moreover, one of the encapsulated drugs (Pal) is fluorescent with 405 nm excitation and emits light between 450 and 500 nm [61]. During illumination, the fluorescence intensity of free Pal decreased, as shown in Figure 8. The conjugation of Cy7 and encapsulation of Pal probably protect the dye and the drug from light-induced factors such as oxygen, oxidizing or reducing agents, temperature rise, exposure time and illumination levels, which may reduce the fluorescence intensity in an irreversible manner.

### 2.9. In Vitro Cell Viability after Exposure to Cy7-P(RGD) and Cy7-P(RGD)/Pal,Alp NCs

**Figure 9:**
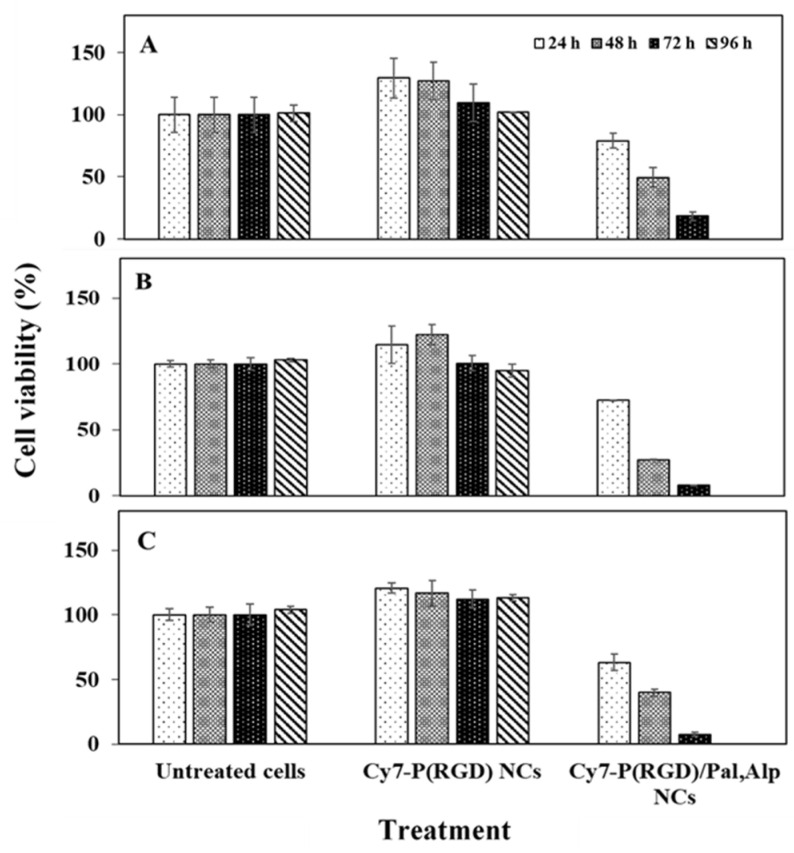
Cell viability of (**A**) MCF-7, (**B**) HCT116 and (**C**) A549 cell lines after exposure to Cy7-P(RGD) NCs measured by XTT assay. Cells (10^4^) were incubated for 24, 48, 72 and 96 h with hollow and Cy7-P(RGD)/Pal,Alp (2.8 µM of each drug) dispersed in water (0.1 mg/mL)). Untreated cells (positive control with medium) were similarly incubated. Bars denote mean ± standard deviation of six separate samples.

In vitro cell toxicity was determined by the XTT assay on three human cancer cell lines– MCF7 (breast adenocarcinoma), HCT116 (colon carcinoma) and A549 (lung carcinoma). Cells were treated for 24, 48, 72 or 96 h with Cy7-P(RGD) hollow NCs (0.1 mg/mL) and Cy7-P(RGD)/Pal,Alp NCs (0.1 mg/mL NCs, 2.8 µM from each drug). Figure 9 exhibits the cell viability levels post treatment. Exposure of cells to Cy7-P(RGD) hollow NCs did not affect the viability after 24–96 h compared to untreated cells, hence the proteinoid NCs are non-toxic. Moreover, the cell viability was amplified (above 100%) after treatment. This can be explained by the uptake of arginine, aspartic acid and glycine as nutrients [62]. While treatment of cells with Cy7-P(RGD)/Pal,Alp NCs showed complete inhibition of cell growth and induction of death after 96 h, it should be noted that the viability exhibited a gradual decrease (from 100%) to 79% after 24 h, 50% after 48 h and 18% after 72 h. These results demonstrate the controlled release of the drugs from the NCs.

### 2.10. Drug Response in a Patient-Derived Xenograft (PDX) Tumor Spheroid Model

**Figure 10:**
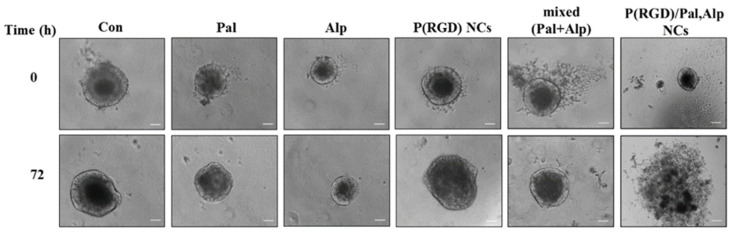
Bright-field microscopic images of patient-derived xenograft (PDX) colon tumor cells after exposure to Pal, Alp, Pal + Alp and hollow and drug loaded P(RGD) NCs. Spheroids were incubated for 72 h. 10× magnification, scale bar = 102 µm.

The efficacy of the drug-loaded NCs was compared to the mixed free drugs by a 3D model of tumor spheroids derived from a PDX model that mimics closely the native tissue microenvironment and the solid tumor better than cells grown in 2D monolayers [45,61]. After generation, the tumor spheroids (diameter of 100–400 µm) were seeded and treated with Pal/Alp or their mixture (2.8 µM of each drug), P(RGD) hollow NCs (0.1 mg/mL), and P(RGD)/Pal,Alp NCs (0.1 mg/mL with 2.8 µM of each drug). As shown in Figure 10, only the drug loaded NCs disassembled the spheroids.

Thus, as expected, the 2D monolayer culture does not account for the diffusion of the drugs in the tumor as there is no concentration gradient in the culture. Moreover, it does not include the ECM around the cells, which functions as a barrier between the cells and the treated drugs, while in the spheroids the layers and ECM network serve as a barrier for penetration of drugs, NCs, etc. [63]. The penetration of the NCs depends on several parameters including size, surface charge, morphology, and ligands. It was reported that nanoparticles with small diameter below 50 nm [64,65,66], positive or neutral charge [67,68] and/or spherical morphology [69,70] increase the ability of the NCs to deliver drugs deep into the inner layer of the spheroids. Without drug (control and hollow NCs), spheroids continued to grow, as they are composed of an outer layer in which cells receive a higher supply of oxygen and nutrients, thus showing a proliferative behavior.

### 2.11. In Vivo Tumor Growth Inhibition in PDX Mice Using Pal, Alp and Mixed (Pal + Alp) Treatment

Two patient-derived xenograft (PDX) models RA-300 and RA-346B were used to study the tumor growth inhibitory effects of Pal and Alp. The RA-300 model was derived from a biopsy of colon cancer (Figure S4A), and the RA-346B model was derived from a biopsy of stomach cancer (Figure S4B). Drug administration started when the average tumor volume was 70 mm^3^ for RA-300 and 96 mm^3^ for RA-346B. Mice were randomized into four groups and treated with vehicle (control group), Alp (25 mg/kg daily orally by gavage), Pal (100 mg/kg daily orally by gavage) and a combination of the two drugs. With RA-300, mice in vehicle group were sacrificed at 14-days post treatment due to excessive tumor growth (1400 mm^3^), while the Pal and combination group reached the tumor volume endpoint only 2–3 weeks later. Mean tumor volumes of RA-300 at day 15 in vehicle control, Alp, Pal and co-treatment were 1381 ± 178, 1051 ± 242, 1094 ± 209 and 530 ± 31 mm^3^, respectively. Mean tumor volumes of RA-346B at day 35 in vehicle control, Alp, Pal and co-treatment were 1488 ± 179, 1586 ± 48, 1655 ± 41 and 767 ± 143 mm^3^, respectively (data are presented as mean ± S.E.M.). Tumor measurement showed that either Pal or Alp could inhibit the growth of RA-300 and RA-346B tumors. Co-treatment showed significant reduction in tumor volume compared with single treatment, with *p* = 0.05 (Pal vs. combined) in RA-300 (Figure S4A) and *p* = 0.002 (Pal vs. combined) in RA-346B (Figure S4B). Both Pal and Alp single as well as combined treatments were well tolerated in the mice. Using these models, it was found that a combination of both targeted drugs, Pal and Alp, had a significant synergistic effect that induces reduction of growth in different types of tumors.

## 3. Materials and Methods

*Materials.* The following analytical-grade chemicals were purchased from commercial sources and used without further purification: D-arginine (CAS number 157-06-2), L-glycine (CAS number 56-40-6), L-aspartic acid (CAS number 56-84-8), sodium chloride (CAS number 7647-14-5), super-pure HPLC water (CAS number 7732-18-5), sodium bicarbonate (CAS number 144-55-8), acetonitrile (CAS number 75-05-8), trifluoroacetic acid (CAS number 76-05-1), and dimethyl sulfoxide (DMSO, CAS number 67-68-5) were obtained from Sigma-Aldrich (Rehovot, Israel). Phosphate buffered saline (PBS) -/- (calcium and magnesium free) (CAS number 02-023-1A), glutamine (CAS number 03-022-1B), penicillin streptomycin (CAS number 03-034-1B), cytotoxicity detection kit (XTT) (CAS number 20-300-1000), and Nutristem HpscxF medium (CAS number 05-100-1A) were purchased from Biological Industries (Bet Haemek, Israel).

Cy7-NHS ester (Cy7-NHS) was obtained from Lumiprobe Corporation (CAS number 15020) (Hallandale Beach, FL, USA). Dialysis membranes (8000 kDa MWCO, CAS number 131198) and bicarbonate buffer (0.1 M, pH 8.4) were purchased from Bio-Lab Ltd. (Jerusalem, Israel). MCF-7, HCT116 and A-549 human cell lines were obtained from the American Type Culture Collection (Manassus, VA, USA) and their medium was purchased from Lonza (Basel, Switzerland). Water was purified by passing deionized water through an Elgastat Spectrum reverse osmosis system (Elga Ltd., High Wycombe, UK). Pal and Alp were purchased from BioTag Ltd. (Kfar Yona, Israel).

*Animals and tissues.* Immunodeficient NRG and NSG mice were acquired from Jackson Laboratories (USA, Canada and Puerto Rico). Cancer specimens were obtained from patients diagnosed and treated at the Davidoff Oncology Department at the Rabin Medical Center (Petach Tikva, Israel). Tissue collection was performed per institutional review board (IRB)-approved protocols with written informed consent from the patients. Cultrex Basement Membrane Matrix, Type 3 (CAS number 3632-001-02), was purchased from Trevigen Inc. (Gaithersburg, MD, USA).

### 3.1. Synthesis of P(RGD) Proteinoid Polymer

Proteinoid synthesis was done similar to the procedure described in the literature [28,48]. For this purpose, 1.67 g each of R, G and D (~5 g total) were introduced into a three-neck flask. The mixture was mechanically stirred (150 rpm) and heated under N_2_ atmosphere to 170 °C for 20 min, producing a highly viscous yellowish to brownish paste. The paste was cooled down to room temperature (RT) and hardened to a glassy mass. After cooling, the residue was extracted by 30 mL of distilled water and lyophilized to yield the crude proteinoid as a resin-colored powder (resembling caramelized sugar) [17]. As mentioned above, the frequency of RGD tripeptides in the random proteinoid sequence was about 13% [28]. The proteinoid polymer was characterized in terms of molecular weight and polydispersity index (PDI) by GPC as described in Refs [24,28].

### 3.2. Characterization of P(RGD) Proteinoid Polymer

The molecular weight and polydispersity index of the dried proteinoid were determined by gel permeation chromatography (GPC) consisting of a Waters Spectra Series P100 isocratic HPLC pump with ERMA ERC-7510 refractive index detector and Rheodyne injection valve (Coatati, CA, USA) with a 20 μL loop (Waters, MA, USA). Samples were dissolved with sodium phosphate buffer through a linear BioSep SEC-s3000 column (Phenomenex, Torrance, CA, USA) at a flow rate of 1 mL/min. The molecular weight was determined relative to standards of proteins: myoglobin from equine skeletal muscle (17.6 kDa), human serum albumin (67 kDa) and bovine plasma fibrinogen (340 kDa) using Clarity Chromatography software (DataApex, Prague, Czech Republic, USA). The optical activity was determined with a PE 343 polarimeter (PerkinElmer Inc., Waltham, MA, USA). All measurements were performed in water at 589 nm and 25 °C.

### 3.3. Synthesis of Hollow Proteinoid NCs

Hollow P(RGD) proteinoid NCs were prepared by a self-assembly process as described previously [26,49]. For this purpose, 100 mg of dried proteinoid polymer were added to a 28 mL glass screw-capped vial; 10 mL of a 0.01 mM NaCl aqueous solution were added, and the mixture was heated to 80 °C on a hot plate. The clear solution was stirred at 250 rpm for 30 min until complete dissolution and left on the plate to slowly cool to RT.

### 3.4. Synthesis of Drug-Encapsulating P(RGD) NCs

P(RGD) NCs encapsulating various concentrations of Pal and Alp were prepared by dissolving proteinoid powder in five glass vials as described above (100 mg in 0.01 mM NaCl solution). A freshly prepared DMSO stock solution containing both drugs (300 µL) was placed on the hot plate along with the NC vials during the constant heating stage (at 80 °C). After 30 min of heating, while still at 80 °C, different amounts of stock solution were added to each vial (see Table 1); Tween 80 surfactant was then added as needed to the cloudy dispersions. The clear yellowish solutions were left to slowly cool to RT (on the plate) and extensively dialyzed through a cellulose membrane (8000 Da MWCO) against distilled water to remove excess drugs [71].

### 3.5. Drug Loading (DL) Measurements by High Performance Liquid Chromatography (HPLC)

HPLC analysis of Pal and Alp was performed using a Hitachi LaChrom Elite system (Hitachi, Tokyo, Japan) equipped with a photodiode array detector, column oven, autosampler, quaternary pump and 100 μL sample loop. The chromatographic separation was performed at 30 °C using an ACE 5 C-18 column (5 µm particle size, 250 × 4.6 mm) under gradient elution conditions. Mobile phases consisting of water-trifluoroacetic acid (999:1, *v*/*v*) (A) and acetonitrile-trifluoroacetic acid (999:1, *v*/*v*) (B) were filtered through a membrane filter (0.22 μm). Gradient elution started at 3% B, increased linearly to 30% B for 5 min, and then increased linearly to 50% B until 15 min. For the next 2 min, the gradient increased linearly to 100% B and stayed isocratic for the next 3 min. For column equilibrium, the gradient decreased linearly to 3% B, and the flow rate was maintained at 1.0 mL/min for 30 min. The injection volume was 5 μL and the column elution was monitored at 340 nm for both Alp and Pal. Chromatogram peak integration and area calculations were performed using EZChrom Elite software version 3.3.2 Service Pack 2 (Agilent Technologies, CA, USA).

For HPLC, drug-encapsulated P(RGD) NCs aqueous dispersion was diluted two-fold with NaCl solution 10^−5^ M containing 0.5% (*v*/*v*) Tween 80 to maintain sink conditions and sonicated in an ice-water bath for 10 min to disrupt the nanocapsules. The sonication caused the proteinoid NCs to disassemble and elute the drugs. The weight of drug in each sample was calculated using the calibration curve as mentioned above.

### 3.6. Conjugation of Cyanine7 NHS Ester

The near IR dye Cyanine7 NHS ester (Cy7-NHS) was conjugated at RT to primary amino groups on the surface of P(RGD) hollow and drug-loaded NCs. To 10 mL of acidic NC solution, 150 μL of 0.1 M sodium bicarbonate were added to obtain pH of 8–8.3 (near the manufacturer recommended range of 8.3–8.5). Cy7-NHS (1 mg in 250 μL DMSO) was added, and the solution was stirred at 150 rpm for 1 h and extensively dialyzed through a cellulose membrane (3500 Da MWCO) against distilled water.

### 3.7. Hydrodynamic Diameter and Size Distribution

The hydrodynamic diameter and size distribution of the NCs were measured with a cryogenic transmission electron microscope (cryo-TEM). Aqueous NC dispersions (3 μL) were loaded on a glow discharged (EmiTech K100 machine, Quorum Technologies, East Sussex, UK) Quantifoil or lacey grid, which was blotted and plunged into liquid ethane using a Gatan CP3 automated plunger (Gatan, Pleasanton, CA, USA) and stored in liquid nitrogen until use. Frozen specimens (samples with vesicles embedded in vitreous ice) were transferred to a Gatan 914 cryo-holder and maintained at temperatures below −176 °C inside the microscope. Samples were inspected with a FEI Tecnai G2 microscope (now Thermo-Fisher Scientific, Waltham, MA, USA) with an acceleration voltage of 120 kV equipped with a cryobox decontaminator. Images were taken using a digital micrograph (Gatan, ver. 1.83.842) with a mulitiscan camera (Gatan, model 794) at varying resolution. The mean diameter was determined by measuring at least 200 particles using the image analysis software AnalySIS Auto version 3.2 (Soft Imaging System GmbH, Münster, Germany). Each experiment was repeated at least three times.

### 3.8. High Resolution Scanning Electron Microscopy (HR-SEM)

The dry diameter and size distribution of the NCs were measured by a high-resolution scanning electron microscope (HR-SEM) model FEI Magellan 400L. A small droplet of aqueous NC dispersion was placed on an aluminum stub and left to dry, followed by coating with iridium. The diameters of more than 200 NCs were measured with AnalySIS Auto image analysis software version 3.2 (Soft Imaging System GmbH, Münster, Germany). Each experiment was repeated at least three times.

### 3.9. Thermal Analysis

The thermal behavior of the proteinoid NCs and the free drugs was determined by thermogravimetric analysis (TGA) with a TGA/DSC 1 STARe system (Mettler Toledo, Greifensee, Switzerland). Samples were heated between 25–700 °C at a rate of 10 °C/min under nitrogen atmosphere.

### 3.10. ζ-Potential Measurements

The surface potentials of the proteinoid NCs were measured in aqueous dispersion at a pH of 7.4 at a concentration of 10 mg/mL using a Zetasizer 3000 ζ-potential analyzer (HSa model, Malvern Instruments Company, Malvern, UK).

### 3.11. Fourier Transform Infrared Spectroscopy (FTIR)

FTIR measurements of proteinoid NCs and free drugs were performed by the attenuated total reflectance (ATR) technique using a Bruker Alpha-FTIR QuickSnap™ sampling module equipped with a Platinum ATR diamond module (Bruker, Berlin, Germany).

### 3.12. UV Absorption Spectra

Absorption spectra of the proteinoid NCs and free drugs were obtained with a Cary 100 UV-Visible (UV-Vis) spectrophotometer (Agilent Technologies Inc., Santa Clara, CA, USA). All measurements were performed in water at 25 °C. Excitation and emission spectra were recorded using a Cary Eclipse spectrophotometer (Agilent Technologies Inc.).

### 3.13. Cy7 Conjugation Yield

Calibration curves of free Cy7 were obtained by measuring the integrals of IR absorbance peaks of standard solutions (0.5–10 μg/mL) in PBS at wavelengths of 700–900. The concentrations of the Cy7-P(RGD) and Cy7-P(RGD)/Pal,Alp NCs were determined by measuring the integral of the corresponding absorbance of a 10 mg/mL dispersion of free Cy7. An estimation of encapsulated material per mg of NCs was determined according to the calibration curves.

### 3.14. Photostability

Aqueous solutions of free Cy7, Cy7-P(RGD) NCs, Cy7-P(RGD)/Pal,Alp NCs and free Pal were prepared to give similar fluorescence intensity; intensities were measured with λex set at 750 nm and λem set at 800 nm except free Pal (λex 400 nm, λem 520 nm). Each sample was illuminated continuously with a xenon lamp and measured over a period of 30 min. Intensity values were normalized for comparison.

### 3.15. In Vitro XTT Cell Viability

An XTT assay based on the ability of the mitochondrial succinate-tetrazolium reductase system to convert the yellow tetrazolium salt XTT (sodium 3′-[1-(phenylaminocarbonyl)-3,4-tetrazolium]-bis(4-methoxy-6-nitro) benzene sulfonic acid hydrate) to orange formazan dye [72] was performed with A549 (lung), MCF7 (breast) and HCT116 (colon) human cancer cells after treatment with NCs. Cells were seeded in four 96 well plates at a density of 10^4^ cells/well in 100 μL of culture medium and grown in a humidified 5% CO_2_ atmosphere at 37 °C. After 24 h at 37 °C, cells were treated with NCs dispersed in water (0.1 mg/mL), Pal/Alp alone (2.8 µM) or Pal,Alp (2.8 µM of each drug). Each of the plates was incubated for a different time at 37 °C (24, 48, 72 or 96 h); 100 μL of XTT solution were then added to each well according to the kit manufacturer’s instructions. The absorbance was read at 490 nm, and the cell viability was determined using the formula in the manufacturer’s protocol [73].

### 3.16. In Vitro Efficacy in Colon Cancer PDX Spheroid Model

PDX colon tumor samples were cut into 2–4 mm^3^ sections after removal of debris (fat and necrotic material) and washed with sterile PBS. Small pieces of tumor were placed in Nutristem HpscxF medium (5 mL) and mechanically dissociated by GentleMACS Dissociator (Miltenyi Biotec, Bergisch Gladbach, Germany). Tissues were completely dispersed and cultured in 25 cm^2^ tissue flasks (LifeGene, Mevoh Horon, Israel) at 37 °C and 5% CO_2_. Viable cells were attached to the bottom and during the following days and weeks, the tumor cells formed colonies that detached and grew as self-assembled spheroids in the medium.

Upon reaching 75 to 100% confluence, one or two spheroids were transferred to 96-well round-bottom ULA plates (Greiner Bio-one, Bad Haller, Kremsmünster, Austria). The culture medium was supplemented with 5% of Cultrex Basement Membrane Matrix and the plates were centrifuged at 300 g for 10 min to improve cell aggregation. After 48 h at 37 °C, the spheroids were treated with proteinoid NCs dispersed in water (0.1 mg/mL per well), Pal, Alp or mixed Pal,Alp (2.8 µM of each drug). Negative and positive control groups were cultured with Nutristem HpscxF medium. After 72 h of treatment, the spheroids in each treatment group and the control were monitored to reveal morphological changes by microscopy and photography. The experiment was repeated twice without medium change. The spheroids were characterized in terms of shape and size using the open-source software ImageJ version 1.52a (National Institutes of Health, Bethesda, MD, USA).

### 3.17. Animal Experiments

All mice were maintained and treated in accordance with both the Rabin Medical Center and the Bar-Ilan University guides for the care and use of experimental animals with approval from the RMC Institutional Animal Care and Use Committee (protocol reference number 14-02-2019).

### 3.18. PDX Model

For establishment of PDX, tumor material was placed in cold DMEM medium supplemented with 10% (*v*/*v*) FBS and 1:100 (*v*/*v*) penicillin/streptomycin antibiotics and maintained on ice until processing. Within 0.5–2 h, tumor fragments were cut into small pieces (approximately 2–3 mm) using sterile surgical instruments. Implantations were carried out into recipient 5–8 weeks old immunodeficient NRG or NSG mice (Jackson Laboratories). Before implantation, the tumor fragments were coated with Cultrex Basament membrane matrix, type3 (Trevigen). Surgery was performed under sterile conditions in a laminar flow cabinet using sterilized surgical instruments. Mice were kept under pathogen-free conditions and received sterilized food and water and libitum. Tumor growth: mice were weighed and inspected 1–2/week for assessment of general condition, and PDX development was assessed by palpation of the site of implantation and measured in two dimensions by electronic caliper. Tumor volume was calculated using the equation (width×width×length)/2. Once tumors reached 1–1.5 cm in diameter, mice were euthanized, and tumors were harvested. Tumor tissue was passaged directly into further generation or storage. Following harvesting, the tumor was washed in sterile saline and mechanically dissociated by GentleMACS (Miltenyi Biotech) and implanted by subcutaneous injection for efficacy studies with drugs.

## 4. Conclusions

This research exhibited proof of concept for nanomedicine-mediated multimodal treatment by co-encapsulation of Pal and Alp within P(RGD) proteinoid NCs. The combined inhibition of both the CDK4/6 and the PI3K pathways provides the rationale for this approach. Pre-clinical studies [44,74] as well as our PDX model showed synergy between Pal and Alp in human colon, bladder and breast cancer, as shown in Figure S4. Furthermore, both controlled release and dose dependence were exhibited.

In the patient, the most common adverse events from combination therapy are neutropenia, diarrhea, leukopenia and fatigue. Other non-hematologic side effects include nausea, vomiting, arthralgia and alopecia. Therefore, one of the goals of this study was to circumvent the problems associated with conventional antitumor drug delivery systems including their non-specificity, severe side effects, burst release, and damaging of normal cells. These drawbacks may be overcome by controlling drug delivery. In controlled delivery systems, the drug is transported to the place of action; thus, its influence on vital tissues and undesirable side effects can be minimized. Drug delivery systems also protect the drug from rapid degradation or clearance and enhance its concentration in targeted tissues, allowing lower doses. In our lab, NCs (defined as having at least one dimension below ~100 nm) were developed, which have great potential as drug carriers.

A P(RGD) proteinoid based on D-arginine, glycine and aspartic acid was synthesized by thermal step-growth polymerization [28], characterized by GPC, and showed high molecular weight and very low polydispersity. P(RGD) NCs co-encapsulating Alp and Pal by a self-assembly process, exploiting the known ability of the RGD sequence to target angiogenesis in the tumor microenvironment by binding the RGD peptide to αvβ3, which is overexpressed by endothelial and/or cancer cells, allowing “active targeting” of the tumor. 

Hollow P(RGD) proteinoid NCs were prepared and characterized in terms of size and size distribution by cryo-TEM and HR-SEM. The wet diameter was 34 ± 5 nm. Maximum encapsulation of the mixed drugs was obtained with 25% drugs/NCs, yielding a wet diameter of 22 ± 3 nm. The loading capacity was determined by HPLC, FTIR and TGA, showing successful encapsulation of approximately 80% of the initial amount. Conjugated Cy7 had better fluorescence stability following long-term excitation than the free dye, which may indicate successful conjugation, protecting it from light-induced factors. 

The effect on cell viability was studied in vitro by an XTT assay in MCF7 (breast cancer), HCT116 (colon cancer) and A549 (lung cancer) cell lines for hollow and drug loaded NCs. Hollow NCs did not have any effect on the cell viability. The results following treatment with P(RGD)/Pal,Alp NCs demonstrated time-dependent release and, moreover, a significant synergistic effect using combined Pal and Alp treatment compared to mono treatment. These NCs thus have great potential as a platform for targeted slow release anticancer treatment. 

Preliminary results of treatment with P(RGD)/Pal,Alp NCs using a 3D in vitro model of PDX colon tumor spheroids illustrated disassembly of the spheroids, which can predict that these NCs would be effective in animal experiment models. Further work includes studying the ability of the NCs for drug release (in vitro controlled release experiment), biodistribution, and in vivo treatment of tumors in PDX mice models such as colon, bladder and breast cancer. Another direction is to evaluate the potential of these NCs to control the release rate and improve their stability by PEGylation of the outer surface. Finally, these NCs hold promise for clinical treatment of various cancers with reduced side effects.

## Figures and Tables

**Table 1:** Components used for synthesis of drug loaded NCs.

Drugs/Proteinoid (*w*/*w*, %)	[Pal ^a^/Alp ^b^] (mg/mL)	Tween 80 (*v*/*v*, %)
1	0.1	0
5	0.25	1
10	0.5	1
25	1.25	3
50	2.5	5

^a^ Solubility of Pal in H_2_O: 4.9 mg/mL (10.1 mM, needed warming). ^b^ Solubility of Alp in DMSO: ≥100 mg/mL (226.5 mM).

**Table 2:** Diameter and drug loading of P(RGD)/Pal,Alp NCs ^a^.

[Drugs]/[Prot.] (*w*/*w*, %)	[Drugs] (mg/mL)	Diameter (nm)		Drug loading (%)	
		Dry	Wet	Pal	Alp
0	0	36 ± 8	34 ± 5	0	0
1	0.2	59 ± 12	30 ± 3	32	25
5	0.5	83 ± 27	28 ± 7	72	88
10	1	87 ± 10	27 ± 5	65	83
25	2.5	113 ± 31	22 ± 3	70	89
50	5	148 ± 36	40 ± 8	72	92

^a^ The hydrodynamic diameter was measured by cryo-TEM; the dry diameter was measured by HR-SEM; the DL was measured by HPLC using calibration curves of standard solutions of Pal and Alp.

## Data Availability

The data presented in this study are available in the article or supplementary material.

## Supplementary Materials

The following are available online at https://www.mdpi.com/article/10.3390/ph14070648/s1, Figure S1: HR-SEM images and histograms of P(RGD) and P(RGD)/Pal,Alp NCs. Wet diameter and size distribution histogram of hollow P(RGD) NCs and P(RGD)/Pal,Alp NCs. Figure S2: FTIR spectra of Alp, Pal, P(RGD) NCs and P(RGD)/Pal,Alp NCs. Figure S3: TGA thermograms of Alp, Pal, P(RGD) NCs and P(RGD)/Pal,Alp NCs. Figure S4: Tumor growth as function of time. In vivo tumor growth inhibition in PDX mice using Pal, Alp and mixed (Pal + Alp) treatment.
