# Supplementary material for: Tumor-Targeted Fluorescent Proteinoid Nanocapsules Encapsulating Synergistic Drugs for Personalized Cancer Therapy
Source: Pharmaceuticals (Basel). 2021 Jul 6;14(7):648. doi: 10.3390/ph14070648 (PMC8308547; doi:10.3390/ph14070648)
Supplement: Supplementary file 1 [file pharmaceuticals-14-00648-s001.zip › pharmaceuticals-1253946-supplementary.pdf]

Supplementary material

Tumor targeted fluorescent proteinoid nanocapsules encapsulating synergistic drugs for personalized cancer therapy

Ella Itzhaki[1], Elad Hadad[1], Neta Moskovits[2], Salomon M. Stemmer[2,3] and Shlomo Margel[1*]

[*] Correspondence: Shlomo Margel, Tel: + 972-3-5318861, E-mail: shlomo.margel@ mail.biu.ac.il

[1] The Institute of Nanotechnology and Advanced Materials, Department of Chemistry, Bar-Ilan University, Ramat-Gan 5290002, Israel

[2] Davidoff Center, Rabin and Felsenstien Medical Center, Beilinson Campus, Petach Tikva 49100, Israel

[3] Sackler Faculty of Medicine, Tel Aviv University, Tel Aviv 6997801, Israel

E-mail: Shlomo.margel@biu.ac.il

elaeli3543@gmail.com

neta.moskovits@gmail.com

stemmer@post.tau.ac.il

eladha300@gmail.com

Figure S1: HR-SEM images and histograms of P(RGD) and P(RGD)/Pal,Alp NCs. Wet diameter and size distribution histogram of hollow P(RGD) NCs and P(RGD)/Pal,Alp NCs.

Figure S2: FTIR spectra of Alp, Pal, P(RGD) NCs and P(RGD)/Pal,Alp NCs.

Figure S3: TGA thermograms of Alp, Pal, P(RGD) NCs and P(RGD)/Pal,Alp NCs.

Figure S4: Tumor growth as function of time. *In vivo* tumor growth inhibition in PDX mice using Pal, Alp and mixed (Pal + Alp) treatment.

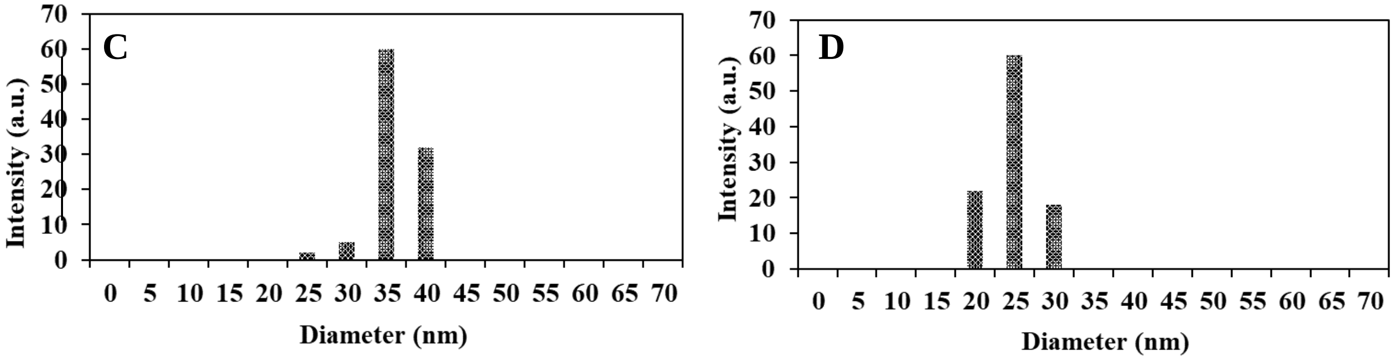

Figure S1: Dry diameter and size distributions obtained by HR-SEM of (**A**) P(RGD) and (**B**) P(RGD)/Pal,Alp NCs. Wet diameter and size distribution histograms obtained by cryo-TEM of (C) hollow P(RGD) NCs and (D) P(RGD)/Pal,Alp NCs.

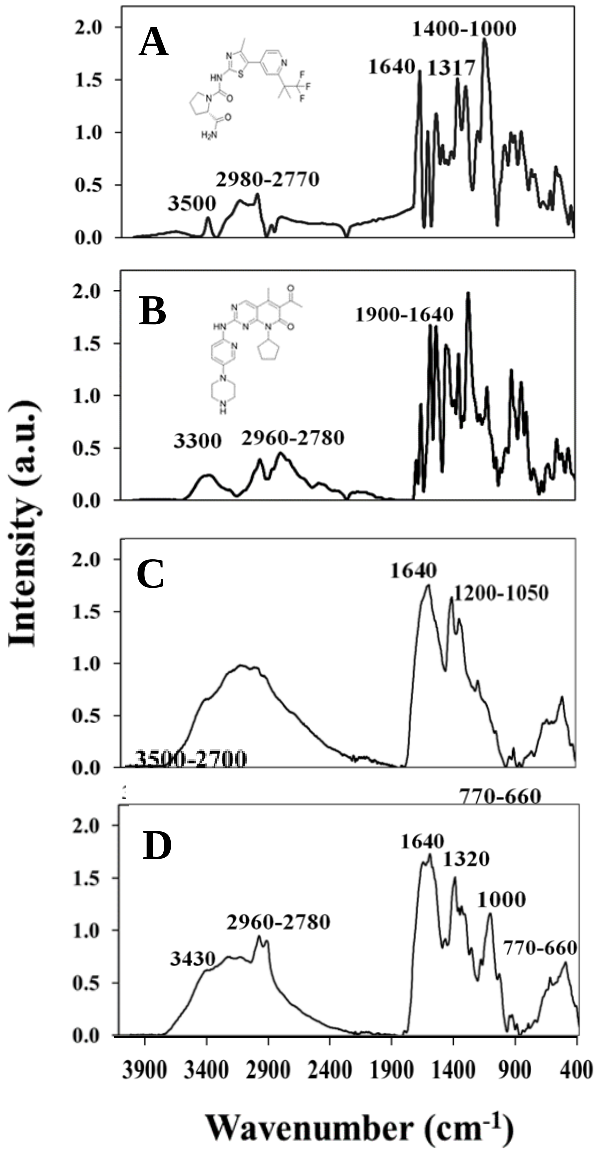

Figure S2: FTIR spectra of (**A**) Alp, (**B**) Pal, (**C**) P(RGD) NCs and (**D**) P(RGD)/Pal,Alp NCs.

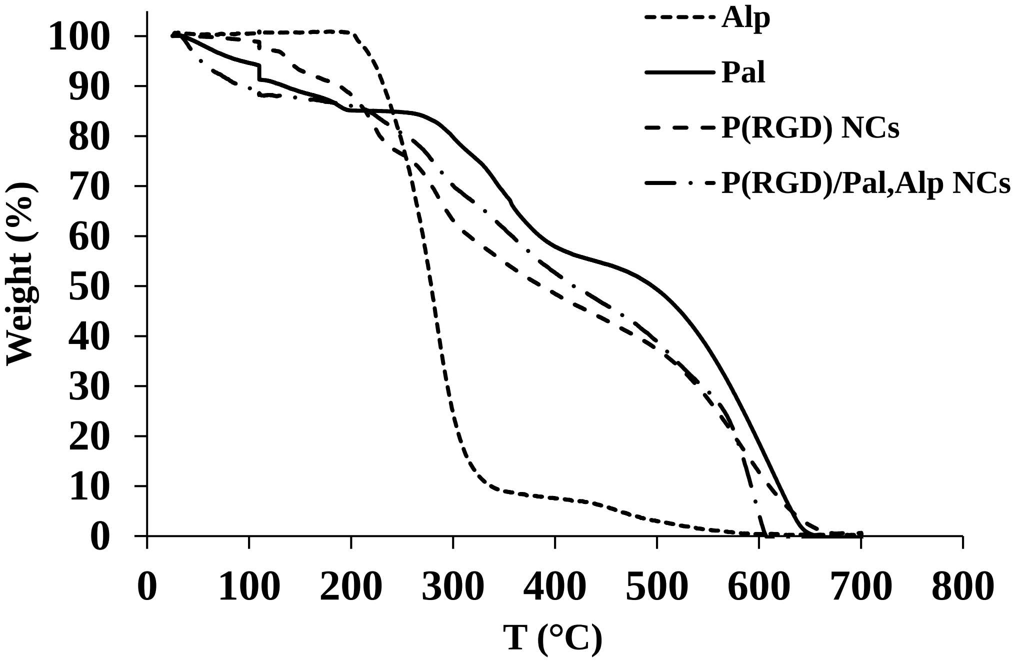

Figure S3: TGA thermograms of Alp, Pal, P(RGD) NCs and P(RGD)/Pal,Alp NCs.

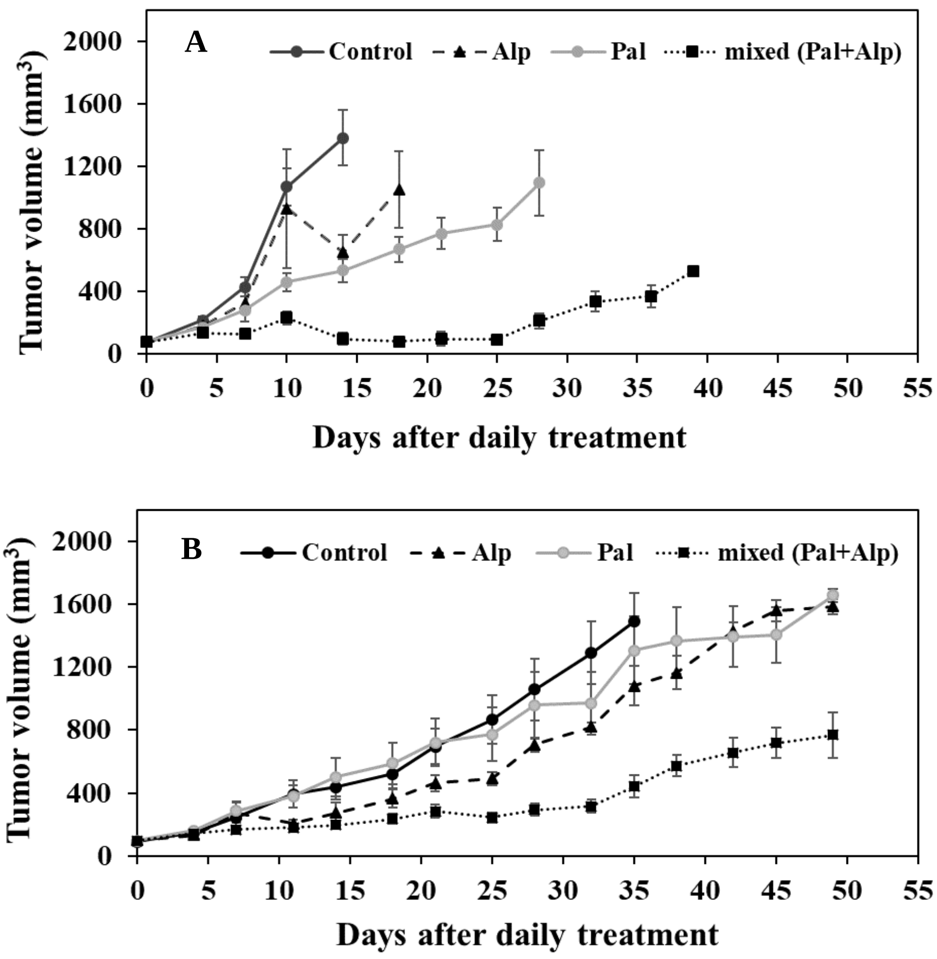

Figure S4: Tumor growth as function of time. *In vivo* tumor growth inhibition in PDX mice using Pal, Alp and mixed (Pal+Alp) treatment. (**A**) RA-300 colon cancer and (**B**) RA-346B stomach cancer. Tumor volumes were measured two times per week, and tumor weight was measured at end of treatment ($n = 4$ to 5 per group at end point, note that some mice were euthanized when tumors exceeded maximum allowable size considering animal ethics).
